# Acquiring the Impossible: Developmental Stages of Copredication

**DOI:** 10.3389/fpsyg.2017.01072

**Published:** 2017-06-28

**Authors:** Elliot Murphy

**Affiliations:** Division of Psychology and Language Sciences, University College LondonLondon, United Kingdom

**Keywords:** copredication, polysemy, paradoxes, semantics, iconicity

## Abstract

Much is known about the acquisition of phonological competence and lexical categories, but there has been substantially less research into word meaning development. In an attempt to contribute to this debate, a group of 24 children aged 4–11 were asked to define a set of words, as were a group of 12 adult controls. The stimuli included both concrete and abstract words, in particular words exhibiting a rare form of polysemy known as copredication, which permits the simultaneous attribution of concrete and abstract senses to a single nominal, creating an ‘impossible’ entity. The results were used to track the developmental trajectory of copredication, previously unexplored in the language acquisition literature.

## Introduction

There are many aspects we can consider when it comes to the question of how children acquire language, with perhaps the most mysterious being the origin of meaning. Hinzen points out in that ‘[t]heories of semantics are about a domain in nature that we do not understand’ (Hinzen, 2011: 419), adding elsewhere that a primary reason is because ‘we haven’t got sense organs for [meanings]’ in the way we do for sounds (2006: ix). Indeed, because of our cognitive ‘scope and limits’ (Russell, 1948), study of human conceptual systems ‘may well fall beyond human naturalistic inquiry in crucial respects’ (Chomsky, 2000: 125). As Hinzen points out in his *Essay on Names and Truth*:

Our minds have managed an analysis of the generative principle of number – an easy case – but they fail badly in the analysis of *house*, which is already much too complex, apparently, and maybe there is not much hope that we will ever succeed for something like *justice*.

Putting this disparity down to ‘principled cognitive limitations,’ he adds that ‘[t]here are reasons for pessimism in this domain… which there may not be in the domain of the mathematical sciences, whose central concepts and modes of understanding are easier’ (Hinzen, 2007: 160–1). Proceeding with the assumption that a naturalistic theory of semantics is possibly beyond our intellectual reach, we can nevertheless inquire through the behavior of children to what extent their semantic and conceptual knowledge appears to have developed. Numerous hypotheses have emerged from centuries of *a priori* and empirical study, such as empiricism, whose adherents claim that semantics originates purely from the data of sense, with a traditional empiricist contention being that ‘the syntactic structure of languages (like everything else) is learned from sensory input’ (Scholz and Pullum, 2006). An opposing set of hypotheses, collected under the banner of nativism, adopts the assumption that experience serves to trigger, and not to form, the meaning of lexical items; assumptions dating back to Plato and Leibniz. Nativists hold that the ability to learn grammar and lexical meaning is hard-wired into the brain and that all human languages share certain structural properties. Indeed, ‘analytic mechanisms of the language faculty seem to be triggered in much the same ways whether the input is auditory, visual, even tactual, and seem to be localized in the same brain areas, somewhat surprisingly’ (Chomsky, 2000: 122). Chomsky has consequently urged that we reverse Aristotle’s dictum that ‘language is sound with meaning’ to ‘language is meaning with sound’ (Chomsky, 2011), with Hinzen (2006) qualifying that, being primarily a thought-system, we should view externalization as a secondary function of language, and ‘communication’ as tertiary, being an even smaller component of language use. To demonstrate more clearly the governing argument of the nativists, we can take the case of Helen Keller, who became death blind after the age of 19 months. She recalls learning the meaning of the word *water* when her teacher placed one of her hands under a water pump and spelled out the word’s five letters with the other: ‘Suddenly I felt a misty consciousness as of something forgotten… I knew then that “w-a-t-e-r” meant the wonderful cool something that was flowing over my hand’ (Keller, 1902/1954: 56). Keller consequently managed to associate a particular concept with the word *water*. In this case, she associated an innate, functional concept to the five letters spelling *water*, defining it not so much through its physical features but rather through its contextual use – something that children and other adults readily do, as the current study will show.

While the acquisition of single words has received much attention (Bloom, 2000; McMurray et al., 2012), the development of more abstract lexical *senses* – such as abstract and concrete – which can be represented through multiple words, has received less attention. More generally, semantic acquisition and development relate to more universal cognitive capacities, such as the ability to conceptualize movement, activities, instruments, locations, recipients, and so forth. By the age of 5 months infants can detect language-relevant data from the environment before selectively responding to certain words, including their own names (Vihman, 1988). After producing their first words at around 10–15 months (Fenson et al., 1994), joint attention appears to be crucial for semantic development, with the infant’s hypothesis that words refer to ‘things’ struggling to maintain coherence when, for instance, an adult says *look* instead of *cat* when pointing to their pet. The first ‘iconic’ stage of lexical development lasts from around ages 1–3 (Özçalışkan et al., 2014), when children are unable to learn abstract words and when they correspondingly realize that objects have names (what we could call the ‘Helen Keller moment’). Before they reach maturity, children assume that words refer to whole objects and not their parts (the ‘whole object assumption’), with the principle of mutual exclusivity dictating that every object can only have one name (Markman, 1994). Infants consequently appear to develop innate perceptual and cognitive strategies for acquiring the meaning of words – an assumption already made in Bruner (1957) and which has been standardly used to support the ‘modularity thesis’ (Fodor, 1983; Gunnar and Maratsos, 1991); the division of sensory objects into ontological categories (e.g., SUBSTANCE and INDIVIDUAL, which help form a child’s intuitive materials-science) is one such well-documented capacity (Soja et al., 1991). In addition, Hespos and Spelke (2004) review experiments which show that, as in speech perception, children do not learn the meanings of words but which distinctions of meaning the language they are exposed to makes, allowing 5-month-old Korean speakers to be sensitive to distinctions marked in their native language but not English. They conclude that ‘the early development of semantic categories parallels the development of phonological categories’ and suggest that semantics ‘evolved to capitalize on pre-existing representational capacities.’ What these pre-existing conceptual capacities are remains largely unknown, with the problem being made no easier by Bloom’s conclusion to *How Children Learn the Meanings of Words*: ‘Nobody knows how children learn the meaning of words’ (Bloom, 2000: 262). All in all, there are reasons to conclude that the development of word meanings relies heavily on non-linguistic conceptual structures.

These ideas are consistent, for instance, with the findings in Gershkoff-Stowe (2002), who showed across two experiments that words which were practiced via production more often than others became more resilient to lexical interference from competing words (in children aged 14.3–17.2 months), and that older children, being more experienced in this process, exhibited proportionally fewer production errors (in children aged 27–30 months). Across all age groups, the source of these productions errors was similar, leading Gershkoff-Stowe to propose the existence of common processing mechanisms used in lexical acquisition. Gershkoff-Stowe (2002: 684) concludes that these results ‘suggest that questions concerning what children know about categories of objects and how they learn the names for things cannot be separated from questions about memory retrieval processes and how conceptual representations are modified with use.’ It appears that these domain-general retrieval processes are heavily involved in language-specific lexical processes of acquisition, much as how non-linguistic conceptual structures contribute to word meaning development.

In what follows, I will review an experiment into the various stages of lexical and metalinguistic proficiency [as Hoff-Ginberg puts it, through elementary school years ‘the internal structure of children’s lexicons changes and becomes more adultlike’ (Hoff-Ginsberg, 1997: 125)]. I will assume that only part of the concept *river* develops before the word is learnt [perhaps its internal geometry, ‘conceptualized as an unbounded line... fattened out by a bounded line... resulting in a surface’ (Pinker, 2008: 180)] in line with the experimental evidence reviewed above; then, with the help of lexicalisation, further complexity is added. This was referred to by Cromer (1974) as ‘the cognition hypothesis,’ a title much too mild today considering the overwhelming evidence. The questions which will concern us most center around the development of metalinguistic awareness in a small sample of children, who range from the ages of 4–11, and how the definitions they give of polysemous lexical items differ.

Unlike much previous work (e.g., Carey, 1978; Soja et al., 1985; Dickinson, 1988), the present set of interviews and analysis do not focus on how children reduce the hypothesis space of a given word’s meaning, since the nature of complex polysemous words does not seem to be tightly constrained by any narrow set of modulated variables in the way that, for instance, the mass/count distinction has often been used to gauge the development of material words, or in the way that the proper/common noun distinction has been used to test the interpretation of unfamiliar objects by 2-years-old (Gelman and Taylor, 1984). No such clear manipulations are available for complex polysemy. Instead, children’s responses to probing questions about the nature and range of polysemous terms were used to track the approximate developmental trajectory of simultaneously conjoining abstract and concrete senses to yield novel forms of polysemy.

The present study focuses on a particular form of complex polysemy, copredication, and all of the eight nominals which the participants were asked to discuss were capable of copredication. These were: *river, word, person, city, book, water, house, ship*. Copredication is standardly defined as ‘a grammatical construction in which two predicates jointly apply to the same argument’ (Asher, 2011: 11). In *He paid the bill and threw it away*, the nominal within the Determiner Phrase (*bill*) takes multiple semantically unrelated predicates, yielding an ‘impossible’ set of semantic relations which could not have a corresponding physical referent. Companies can be *demolished* but also *criticized*, cities can be *sunny, upland* and *liberal*, bills can be *paid* and *folded*, and newspapers can be *read, held, sued, located outside the city* and *unable to offer John a job.* Mixing an abstraction with the concrete appears to be part of the productive nature of lexical semantics, but the developmental properties of these phenomena are unknown. One of the core diagnostics for copredication is anaphora of the kind found in the *bill* example, where the pronoun triggers a distinct sense of the nominal (Asher, 2011). Polysemous words are single phonological forms coding multiple semantically *related* meanings (e.g., *run, key*). This is in contradistinction to homophones, which are single phonological forms coding multiple semantically *unrelated* senses, e.g., *pupil*. Copredication, in contrast, involves the attribution of semantically unrelated senses to a single (and not multiple) polysemous word to derive its meaning, such as in *book* (whose informational content can be read and whose physical features can be touched) and *lunch* (which can last for a given duration whilst also bearing particular flavors, again yielding clear semantic type-theoretic contradictions). Basic polysemous nominals do not permit copredication (e.g., #*James Joyce is on the top shelf and he’s had a haircut*, which does not permit simultaneous reference to an individual and his creative works), and only a small number of nominals allow it – typically, *book*-type, *lunch*-type and *city*-type. This can often lead to certain paradoxes, explored in the present study: For instance, since *book* can host semantically distinct senses, this can lead to a situation in which *John read and burned every book in the library* can result in different numbers of books being read and burned, since a library can contain multiple physical copies of a single informational book.

Even though the developmental properties of copredication are unknown and it is possible only in a relatively limited range of nominals, the basic operations of semantic conjunction (Gotham, 2015) are all that is needed to generate it. Concerning simultaneously abstract and concrete objects, Elbourne (2011: 26) comments: ‘We still have to explain how it is that we can say something apparently straightforward and true, like [abstract and concrete books], but using self-contradictory concepts. I am not aware of any work on this.’ A number of researchers (Asher, 2011, 2015; Jezek and Vieu, 2014; Gotham, 2015) have since cleared the ground for a *formal* account of certain aspects of copredication, typically focusing on numeric quantification and telicity – but much terrain is yet to be covered, such as the processing properties of copredication (see Murphy, 2017 for initial directions) and, as addressed here, its developmental properties.

While they do not address copredication, Srinivasan and Snedeker’s (2011) study of the representation of polysemous and homophonous meanings in 4-year-old children is perhaps the closest in the literature to the present experiment. They raise the possibility that the generative operations or lexical properties which yield polysemy might support children’s representations of polysemy. They show that 4-years-old can understand both the concrete and abstract meaning of *book*, and therefore argue that polysemous senses rely on a common representational base from early in development. Polysymous senses are either stored independently in the lexicon (termed the List Model by Srinivasan and Snedeker) or share a common representational base (the Generative Model, or One Representation Hypothesis). They taught a group of 4-years-old a novel label (such as *blicket*) that corresponded to a single known meaning of a polysemous word (such as the information sense of *book*). Their results suggested that children can readily comprehend extended uses of these novel labels to another sense of the same polysemous word, suggesting that the senses share the same lexical entry, or representational base (see Srinivasan and Snedeker, 2011 for further details). They concluded that polysemous words seem to be acquired not by relating two distinct senses, but rather through ‘foundational properties of the mental lexicon or conceptual system’ (2011: 250). This would also support the finding that polysemous words are recognized faster than homonyms or monosemes due to their richer semantic representations (Rodd et al., 2002).

Philosophers typically argue that ‘concrete’ things exist contingently (such that an individual table or pencil could exist at one moment and cease to exist at another), whereas ‘abstract’ things exist necessarily (e.g., Moltmann, 2013), without acknowledging that both things can exist side by side in cases such as *The book was funny but weighed a ton*. Meanwhile, developmental psychologists have argued that concrete and abstract meanings emerge at distinct maturational stages (Bergelson and Swingley, 2013), but the question of when copredicated structures gain their full semantic complexity has not yet been addressed. Does the concrete sense of *book* emerge before the more abstract (and also more commonly used and accessed) sense? The contingency doctrine does not fare too well when pitted against books, lunches and cities. Much remains unexplored, and it is my intention that this brief study will serve as an entry point into this topic. For instance, while it is known that proverbial and other forms of figurative knowledge become more robust in pubescent years (Nippold, 2000), the developmental trajectory of paired abstract-concrete polysemous concepts has not been investigated.

With respect to a child’s knowledge of copredication, it seems that there are only three logically possible developmental stages that children can exhibit: Knowledge of *one* polysemous sense of the nominal, knowledge of *multiple* polysemous senses of the nominal (typically limited to two, but sometimes more in cases such as *newspaper* and *city*), or knowledge of multiple polysemous senses of the nominal and the *interactions* these senses permit (**Table 2**). For instance, if a child simply understood both polysemous senses of *book* but did not explicitly relate them, they would be unable to understand that two copies of the same informational *book* could be taken out of a library by different people and be defined as identical, with the criterion of identity relying on the INFORMATION sense at the expense of the PHYSICAL OBJECT sense. These forms of semantic conflict are at the heart of copredication, and it was the goal of the present study to explore their developmental basis.

At this point, a number of possible hypotheses seem possible. In support of the List Model, we would expect to find no close correlation between the emergence of any discrete polysemous senses and the subsequent development of copredication. Under this hypothesis, these two developmental processes should be remote. In support of the Generative Model, we would expect to find not only a close correlation between the emergence of polysemous senses, but we would also expect the development of copredication to emerge soon after the acquisition of multiple senses of a single nominal.

## Materials and Methods

### Participants and Procedure

Using an audio recording device, 24 children aged 4–11 (12 male) answered a series of questions exploring the definitions of certain concrete, abstract and complex polysemous words. The children were at Bishop Martin C.E. Primary School, in Liverpool, England. In the discussion below, all of the children’s names have been changed from their real names. The children were sat with their parent or guardian in a sound-proof room, and were told to respond to the questions as comprehensively as possible. The study followed the guidelines in the University of Nottingham’s Code of Research Conduct and Research Ethics, such that each child’s parent or guardian signed a consent form. Interviews lasted on average 8 min (range: 7–10), with the first few minutes being non-experimental questions to ease the child into the process and for them to familiarize themselves with the Question and Answer procedure. This procedure was sufficient in ensuring that the children (in particular, the youngest ones) were able to cope as best they could with the questions, and clear answers were given by all participants to all experimental questions. The experimental questions put to the children, discussed below, either asked them to define a particular word, or to discuss its application in a given scenario and whether this application was appropriate. The interviewer had no personal connection with any of the children. At no point were the children presented with any visual or auditory stimuli and they were asked to answer the questions based purely on their intuitive reactions. All subjects gave written informed consent in accordance with the Declaration of Helsinki. The protocol was approved by the School of English.

### Stimuli

In order to take into account the children’s different pragmatic knowledge due to their age differences, it was ensured that the questions posed to them were structured in a clear, basic form requiring no additional knowledge beyond what was contained within the sentence in order to answer it sufficiently. An example question posed to the children was ‘What is a river?’ with a follow-up question probing the nominal’s polysemous senses being ‘What if the river froze over and cars started driving on it?’. The children were given as much time as they needed to answer the question, and no further prompting was given to them which might prime one or another sense of the nominal (or indeed any possible relation between senses). Since overt productions were solely relied upon, it is only possible to make positive inferences from the data. The children were asked 8 core questions concerning the nominals, with some additional ones being added to explore the different polysemous senses. The children’s answers were clear with respect to which sense of the polysemous nominals they were referring to, such they could not simulatensouly be referring to both abstract and concrete senses of *book* if they defined it as ‘green and big.’ Questions were only repeated if the child requested them to be. Responses to the same questions were also recorded from 12 adult controls (7 female, age range: 24–55), who bore no personal relation to the children and who also signed the appropriate ethics form. What follows are reports of some of the responses given by a selection of the children (see the Supplementary Material for full experimental transcripts selected from 5 of the 24 participants).

## Results

The responses were analyzed based on the number of senses each child demonstrated an understanding of, as reported in **Tables 1, 2**, permitting the recording of a clear developmental trajectory for each polysemous nominal. No effects of gender were revealed by a one-way analysis of variance, and so the results were collapsed over this variable. The dependent variable was the range of senses the children discuss in their definition of copredication. As **Table 2** demonstrates, the children only began to discuss both abstract and concrete senses for all nominals at around age 7. There was also a gradual age-based increase in the number of senses each child demonstrated an understanding of for each nominal. A Kruskal–Wallis *H* test showed that there was a statistically significant difference in these sense comprehension scores between the different ages, crossing Age (4–11) with Sense Number (1 sense, 2 senses, or an interaction between the senses, coded as 3) across each nominal: χ^2^(7) = 20.296, *p* = 0.005. In addition, five of the eight nominals displayed significantly different rates of sense production across the eight age groups (from 4 to 11) (*p*-values are corrected for multiple comparisons): *ship* [χ^2^(7) = 19.190, *p* = 0.008], *book* [χ^2^(7) = 17.889, *p* = 0.012], *city* [χ^2^(7) = 17.907, *p* = 0.012], *person* [χ^2^(7) = 17.090, *p* = 0.017], and *word* [χ^2^(7) = 17.895, *p* = 0.012]. Since an understanding copredication for *river* was exhibited by every child, no significant differences were found. The combination of senses the children produced for *water* was not chronologically variable enough or of sufficiently increased complexity to yield a significant difference [χ^2^(7) = 11.317, *p* = 0.125]. Finally, although the children began to produce an understanding of copredication in *house* at age 11, the progression from single sense to multiple sense understanding and onto copredication was not chronologically varied enough to yield significance [χ^2^(7) = 10.984, *p* = 0.139]; compare the development of *house* with *ship* in **Table 2**.

**Table 1:** Means and standard deviations for the 8 nominals investigated.

*Nominal*	Mean	Standard Deviation
River	3.00	0.000
Word	2.42	0.830
Person	2.17	0.637
City	1.75	0.847
Book	1.71	0.751
Water	1.63	0.824
House	1.38	0.647
Ship	1.13	0.338

*The means are based on the extent to which the children exhibited an understanding of copredication, with ‘1’ indicating 1 sense, ‘2’ indicating 2 senses, and ‘3’ indicating an understanding of possible relations between these two senses, with the range of understanding being from 0 (exhibiting no understanding of any sense) to 3 (copredication). For instance, all of the children understood that the abstract and concrete senses of river can relate, whereas few of them expressed an understanding of the multiple senses of water and their possible relations.*

**Table 2:** Coding the responses of the 24 children.

Participant	River	Word	Person	City	Book	Water	House	Ship
*Samantha (4;8)*	**A + C**	C	A, C	**A + C**	A, C	A	A	C
*Holly (4;5)*	A + C	C	A	A, C	C	A	A	C
*Rosie (4;5)*	A + C	C	A	A	C	A	A	C
*Sam (5;8)*	A + C	C	A	A	A	A	A	C
*George (5;11)*	A + C	C	**A + C**	C	A, C	A	A	C
*Jennifer (5;4)*	A + C	C	A, C	A, C	A, C	A	A	C
*Michael (6;8)*	A + C	C	A, C	A	C	A	A	C
*Imran (6;6)*	A + C	C	A, C	A	A	A	A	C
*Jane (6;2)*	A + C	C	A, C	A, C	A	A	A	C
*Poppy (7;4)*	A + C	C	A, C	A + C	A, C	A	A	A, C
*Kate (7;2)*	A + C	C	A, C	A + C	A, C	A	A, C	A, C
*Rachael (7;7)*	A + C	C	A, C	A + C	A	A	A, C	C
*Ben (8;4)*	A + C	A, C	A, C	A + C	C	A	A	A, C
*James (8;7)*	A + C	C	A, C	A + C	A	A	A, C	A, C
*Sally (8;1)*	A + C	A, C	A, C	A + C	A	A	C	A, C
*Andrew (9;7)*	A + C	A, C	A, C	A + C	A, C	A	A, C	A, C
*Greg (9;9)*	A + C	A, C	A, C	A, C	C	A	A, C	A, C
*Kylie (9;4)*	A + C	A, C	A, C	A + C	A	A	A	A
*Libby (10;7)*	A + C	**A + C**	A + C	A + C	**A + C**	A, C	C	**A + C**
*Tom (10;5)*	A + C	A	A + C	A + C	A + C	A	A	A, C
*Darren (10;7)*	A + C	A + C	A + C	A + C	A + C	A	A	A, C
*Lily (11;4)*	A + C	A + C	A + C	A + C	A + C	A, C	**A + C**	A + C
*Paul (11;6)*	A + C	A + C	A + C	A + C	A + C	A, C	A	A + C
*Adam (11;5)*	A + C	A + C	A + C	A + C	A + C	A	A + C	A + C

*Each child’s age is indicated to the right of their name in standard (Year;Month) format. ‘A’ and ‘C’ refer to the Abstract and Concrete senses of each polysemous nominal in the top row. If the child exhibited understanding of only one of the senses, this is denoted with ‘A’ or ‘C.’ If they exhibited understanding of both senses, this is denoted with ‘A, C.’ Finally, if they exhibited understanding of possible relations between these senses, this is denoted with ‘A + C.’ The black ‘A + C’ denotes the first developmental occurrence of the copredication.*

These results will be discussed based on the type of polysemous nominal discussed. The development of word meaning and metalinguistic awareness of participants can be aptly characterized by certain stages. I will here primarily discuss the responses of five children of particular interest, mainly because they are representative of their age group but also because each of these children were developmentally the first to exhibit understanding of at least one of the copredications examined (**Table 2**). In the current sample, when asked ‘What is a word?’ the responses of the youngest participant, Samantha (4), demonstrate little metalinguistic awareness like the rest of her cohort, defining a word simply as ‘A sound.’ She consequently associates her favorite word with her favorite referent or concept (‘Love’), being unable to consider and evaluate the phonetic features of words (defined as ‘C’ in **Table 2**, for Concrete sense only). George (5) also provides a purely functional definition of *word* (‘It’s like a cat’), unlike the older Kate (7), who identifies words as being inside dictionaries. Kate, with her developed metalinguistic competence, also recognizes *blah blah* as ‘not a real word.’ She does, at the same time, say her favorite word is ‘red,’ ‘Because I like the color’; a somewhat less developed response than Libby (10), who justifies her favorite word (‘Love’) by citing its multiple meanings (platonic, romantic, etc.). Samantha (4) views words as strictly iconic, responding to the question ‘What would happen if we started calling a radiator a toothbrush?’ with ‘You’d be using a radiator for a toothbrush,’ believing the word *radiator* is an inherent feature of the object itself. She does, however, understand the subordinate category *fruit* – knowledge revealed by her use of the final pronoun in her definition of the concept as ‘It’s juice and you can eat them.’

Overextensions in categories were not encountered in the current sample, corroborating much research into the rarity of such occurrences (Rescorla, 1980). A typically functional definition of the basic level word ‘water’ is also given by Samantha (4) (‘Something that you can drink’), with only Libby (10) being able to situate it into a superordinate category *liquid*. Samantha’s linguistic development appears fairly representative of the iconic stage, though her articulatory skills are perhaps more advanced. The older Libby, being at the transitional stage of semantic development, recognizes that calling a radiator a toothbrush would be ‘silly really, because if you use a radiator as a toothbrush… it’d be weird because it’s not a brush and it’s not for your teeth,’ understanding the absurdity of swapping two concrete identities (she acknowledges that the definition of an object is largely based on ‘what you use it for’), though still viewing words as iconic signifiers. Lily (11) seems to have outgrown this tendency, replying that ‘People would think it’s something else until people got used to it.’

Samantha’s (4) handling of simultaneously concrete and abstract features places her more in the functional/perceptual stage than the first stage, since she recognizes that the definition of a river is based more on intentional considerations than material constituency, agreeing with every other participant that a river would still be the same river if its flow were to be reversed, though also recognizing that if the river froze over and cars started driving on it, ‘It would be a road,’ exhibiting a competence understanding of the range of available copredications with respect to *river* (termed ‘A + C’ in **Table 2**). The definition of a *river* or *tree* resides in ‘a Sympathy of Parts’ directed to ‘*one common End*,’ the Earl of Shaftesbury Ashley-Cooper (1711/2001: 349–350) argued, stressing the importance of human interests and concerns also noted by Kate (7) (‘It’s like where people can go fishing, and made of water’), but not George (5).

Adding also to Hobbes’s (1659/2010) investigations into the meaning of *river*, we might add that the flow of a river can be reversed and diverted, divided into subsequently converging streams, and changed in innumerable ways and yet remain the same *thing* – something that Lily (11) is quick to point out, along with Libby (10), since ‘it wouldn’t be rebuilt with concrete.’ She is also the only participant to know about H_2_O, arguing that water is ‘just a different name’ for it, since to her they are identical. Folk-scientific concepts (*water*) and various assemblies of molecules (H_2_O) yield separate concepts with different properties, though children (and many adults) are innate ‘externalists,’ believing that the meaning of, for instance, *water*, is determined by the physical properties of the thing is can be used to refer to (recall that Helen Keller learnt the meaning of *water*, not H_2_O), not realizing that a *thing* is just as obscure a notion as a *sound* or *word*. The sheer prominence of externalist intuitions such as ‘water = H_2_O’ seems to run counter to Clark’s principle of contrast, which stipulates (correctly though controversially) that different words have different meanings (Clark, 1993).

Moving to another form of copredication, Samantha (4) seems to know what a *house* is, though she struggles in understanding what it would mean to ‘paint’ it (a highly intricate word meaning something like ‘intentionally covering a surface with paint’), possibly because learning nouns relies less on context than learning verbs, with the latter requiring a complex understanding of intentional motion, geometric structure, causality, temporality, and more abstract notions (Tomasello and Barton, 1994) which often require particular physical contexts to grasp properly. This is also likely due to the noun-rich nature of early language acquisition (Nelson, 1973). She has, however, clearly moved beyond context-bound word use, discussing her decontextualised semantic knowledge of houses when there are none in sight (Barrett, 1995). All of the participants also demonstrated an understanding that painting a house brown would result in the exterior surface being painted (confirming the intuition in Chomsky, 2000, which claims this to be the case, in contrast to Mukherji, 2010), since a designated exterior is imposed on the object by its semantic features in terms of intended design. The children were also given the following scenario (Supplementary Material): ‘If I put lots of books in my house and invited people to take the books, could it be a library?’. Only the oldest children – Lily (11) and Adam (11) – produced abstract, functionalist responses to this question, agreeing that the same physical building could assume a distinct identity pending human interests and concerns, with the younger children’s conception of *house* being primarily based on physical features.

Before the interview, the children were asked if they had any siblings; if so, they this additional question concerning swapping their sibling’s name was added to further explore the child’s understanding of personhood. The word *sister* was understood by the younger participants to be inherently associated with a referent, with Samantha (4) believing that if she and her sister swapped names ‘I would be 9 because my sister’s 9, and she would be 6.’ George (5) and the older children believe that trading names does not result in trading identities (‘She would call me a different name’). Locke, it seems, was right in arguing that ‘[person] is a forensic term appropriating actions and their merit; and so belongs to intelligent agents, capable of a law, and happiness, and misery’ (Locke, 1689/1836: 234). When presented with the fairy tale of a prince who turns into a frog before being turned back, Samantha (4) mixes the concrete identity of the frog’s body with the personal identity of the prince’s self: ‘He’s a prince-frog, and when the girl kisses the prince he turns back.’ The slightly older George (5), however, argues that ‘No, he’s a frog,’ demonstrating a less refined concept of personhood, though clearly an existing one, with the use of ‘he’ presupposing an understanding of *self*; what Strawson (2009) believes to be the most complex human concept. The three older children argue affirmatively, with Libby (10) agreeing with Locke that the core feature of personhood is ‘personality’ and consciousness.

When asked whether Liverpool would still be ‘Liverpool’ if it were rebuilt on the other side of the country, again George (5) says ‘No.’ His grasp of the abstract features of cities appears to develop later than his classmates, with only Libby (10) agreeing with him, but not for the same reasons, since she questions Liverpool’s changing identity not because she disagrees with the arbitrariness principle, but because she thinks deeper into the social ramifications of moving a city. George (5) also gives a functional/perceptual definition of *book*, and (perhaps surprisingly) believes that if he and his sister were to take two copies of the Bible out of the library they would be taking out the same book, with his understanding of the abstractness of concrete objects being more developed than his concepts of personal identity. The opposite seems to be the case with Libby (10), whose exhibits an understanding of the concrete features of *book*, but not its abstract (INFORMATION) features. Lily (11), however, believes the same book is taken out, stressing the importance of authorial – and hence *personal* – intention (‘Same stories written by the same people’). When questioned about the different forms the information may take, she answers ‘It’s still a book. You can download it on your laptop.’ This struggle between personal and physicalist concerns echoes in the definition of *ship*, and whether a ship owned by an individual’s father remains identical according to a particular sense even when its original material constituency is altered and the old parts are constructed to make a second ship. Kate (7) believes ‘The new one’ to be her father’s ship, ‘because it would be much cleaner.’ Libby’s (10) understanding of the ship’s identity is subordinate to the identity of the ship’s parts, owned by her father (‘because it’s your parts’), while Libby’s (10) concern for ownership goes beyond this, believing both would be her father’s ships ‘because that’s what the definition of “yours” is.’

The responses of the 12 adult controls were fairly similar to that of the older children, giving functional definitions of *book* and *river* while also intuitively equating folk-scientific concepts (*water*) with physical entities (H_2_O). The semantic development of the 10- to 11-years-old was correspondingly adult-like, with only the younger 4- to 5-years-old struggling to categorize concrete objects. The adults also used more nominals than the children. But according to the present sample, Liverpool is still deemed *Liverpool* even after it has been assigned a different spatiotemporal location by all children aged 10 and above. All adults displayed an understanding of copredication in all of the nominals, with the exception of *water*, whose ‘impossible’ structure (from the perspective of folk physics) was never grasped by any of the participants. Every participant scored ‘2’ for *water*, understanding its multiple senses but never grasping their possible relations. Across all the other nominals, each adult scored ‘3’.

## Discussion

As **Table 2** indicates, only the ages of 4–5 and 10–11 involve the introduction of new copredications into the children’s answers out of the eight nominals investigated. The period between aged 5 and 10 seems to involve the acquisition of multiple polysemous senses with respect to *word, house, book* and *ship*, and it is not until aged 10 and onward that the semantic relations between these senses can be properly understood – and in the case of *water*, the children (and indeed the adult controls) never developed this level of understanding. Given the types of nominals which fit into each of the two central stages (4–5 and 10–11), we can draw some potential theoretical conclusions. Although it might initially appear paradoxical, it seems that the more complex nominals involving the highest number of semantically complex polysemous senses (*person* and *city*) permit copredication years in advance of nominals with only two polysemous senses (*word, house, book* and *ship*). A *city* can be an institution, a location, a collection of physical buildings, or a government/polity, while a *person* can be a conscious self, a physical body, or metonymically representative of some body of work. In contrast, the other nominals can only host two senses (e.g., *book* is composed of INFORMATION and PHYSICAL OBJECT senses). It may be that because the more complex nominals have a greater number of senses there is consequently a greater statistical likelihood of any child acquiring (and indeed relating via copredication) at least two of these senses, whereas in the case of less complex nominals like *ship* or *word* the child has to develop a full understanding of the nominal before copredication can be licensed. Finally, with respect to the question of polysemous sense representation, the present results can be argued to support the Generative Model (or One Representation Hypothesis), with the idea that distinct polysemous senses share a common representational base seemingly being supported by the finding that understanding of copredication emerges soon after understanding of the discrete polysemous senses does, such that the common representation can be used to support an understanding of the possible semantic relations between different senses. The only notable exception to this trend is *book*, with the relatively large gap between acquisition of both senses and acquisition of copredication for this nominal likely being explained by the fact that the particular copredication investigated in the present study involved complex numeric quantification operating discretely over one but not both senses, recruiting certain non-lexical mathematical processes to compute the copredication.

My interpretation of this brief survey is that it suggests that knowledge of polysemous semantic features has clear developmental stages, with basic forms of copredication emerging at the point of production (though not necessarily comprehension) around the age of 4, but other, more complex forms not emerging until around age 10. Even when prompted to think about the distinct senses of complex polysemous words like *book* and *river* and consider possible relations between them, children below the age of 10 exhibited no ability to comprehend any possible interactions between semantically distinct yet lexically related senses. With these brief assumptions, we find that a child’s innate semantic knowledge provides rich perspectives through which the environment world may be interpreted.

It was discussed above how the development of word meanings appears to rely on non-linguistic conceptual structures. Yet, this is not to say that certain cognitive primitives responsible for complex polysemy (e.g., the discrete polysemous abstract and concrete senses which generate the meaning of *book*) simply appear as a result of maturational processes and do not require experience in order to be developed. It may well be that certain nominals require broader forms of perceptual experience in order for the relations between senses to be developed and matured. For instance, due to the close physical relation between the two senses of *river*, it is likely that complex polysemy is acquired much earlier for this nominal than it would be for nominals which permit sense relations which exhibit much more physically distant connections, as in *newspaper* (where the institution is contrasted with the printed paper).

The environment doubtless has some effect in determining word meaning (indeed, material constitution may be a more determining factor for *river* than for *book*), but the nature of a child’s lexicon also appears to develop based on quite independent factors (Goodman et al., 1998; McMurray et al., 2012). For instance, McMurray et al. (2012) propose that a dynamic associative model for word referent learning in which denotational selection is simulated as a competition between competing referents, accounting for a number of independent findings about delays in expressive compared to receptive vocabulary and word acquisition during high levels of referential ambiguity. This may in turn account for the delays in copredication licensing documented here, since by their very nature copredications often involve unusually disparate referents crossing core conceptual categories, where *newspaper* can denote an institution which can be sued or a webpage with textual information. The nature of these delays in turn suggests that these forms of dynamic associative models require development, through which the robustness of a child’s associative model can manage an enlarged lexicon.

If these assumptions are along the right track, a suitable follow-up experiment would be to submit a similar group of children to a course of semantic training such that they are exposed to a range of polysemous nominals exhibiting both abstract and concrete senses. In particular, training would involve presenting children with a range of simple, easily comprehendible example scenarios demonstrating various possible copredications as a means of exposing them to the numerous roles distinct polysemous senses can play in delimiting the identity of certain objects. It could also be tested whether exposing children to one sense of a complex polysemous nominal (e.g., the INSTITUTION sense of *newspaper* over its INFORMATION sense) strengthened their understanding of subsequent copredications more than another sense. If there is a close match between the performance scores documented here and semantic competence, we would expect that this training would have little impact on performance with respect to the possibility of comprehending particular copredications. However, it could also be the case that the time which children take to grasp a particular copredication is modulated by the discrete sense(s) they are exposed to, such that, for instance, they are quicker to develop an understanding of *newspaper* copredications if exposed more to INSTITUTION-based scenarios than INFORMATION- or PHYSICAL OBJECT-based ones. Indeed, there may well be a correlation between the type of sense emphasized during training (e.g., an abstract sense) and the rate of acquisition and depth of understanding exhibited as a result. This topic will have to be left to future research.

## Author Contributions

The author confirms being the sole contributor of this work and approved it for publication.

## Conflict of Interest Statement

The author declares that the research was conducted in the absence of any commercial or financial relationships that could be construed as a potential conflict of interest.
