# Supplementary material for: Acquiring the Impossible: Developmental Stages of Copredication
Source: Front Psychol. 2017 Jun 28;8:1072. doi: 10.3389/fpsyg.2017.01072 (PMC5487451; doi:10.3389/fpsyg.2017.01072)
Supplement: Supplementary file 1 [file Data_Sheet_1.DOCX]

Supplementary Material

Acquiring the Impossible: Developmental Stages of Copredication

Elliot Murphy*

*** Correspondence:** elliot.murphy.13@ucl.ac.uk

# Supplementary Data

Below are interview transcripts involving 5 out of the 24 children who took part in the experiment. The children have been anonymized and their ages in the Year;Month format.

Samantha (4;2):

Q: What’s a river?

A: A line of water.

Q: Imagine a river which suddenly starts to flow backwards. Is it the same river?

A: Yes.

Q: What if the river froze over and cars started driving on it?

A: It would be a road.

Q: What’s a word?

A: A sound.

Q: What’s your favourite word?

A: Love. It’s nice.

Q: What would happen if we started calling a radiator a toothbrush?

A: You’d be using a radiator for a toothbrush [laughs].

Q: What would happen if you swapped names with your sister?

A: I would be 9 because my sister’s 9, and she would be 6.

Q: If a prince in a fairy tale gets changed into a frog by a wicked witch, and then changes back again after he gets kissed by his sweetheart, is the frog still the prince?

A: He’s a prince-frog, and when the girl kisses the prince he turns back.

Q: If England was destroyed in a big explosion, and Liverpool was rebuilt on the other side of the country, would it still be Liverpool?

A: Yes.

Q: What’s a book?

A: It’s something that you can read in and there’s loads of words in it.

Q: If you and your sister go to the library and take out two copies of the Bible, did you take out the same book?

A: No because they’re built differently.

Q: Imagine your Dad has a ship and he’s sailing around the ocean, and every day a part of the ship falls off and a new part has to be put on it. After a few weeks every single piece of the ship has been replaced, and all the old pieces are collected and put together to make another ship. Which of the two ships is your Dad’s?

A: The one before it was fixed.

Q: What’s fruit?

A: It’s juice and you can eat them.

Q: What’s water?

A: Something that you can drink.

Q: What’s a house?

A: Where you live.

Q: If I put lots of books in my house and invited people to take the books, could it be a library?

A: It’s not a library if you live in it.

George (5;11)

Q: What’s a river?

A: Water.

Q: Imagine a river which suddenly starts to flow backwards. Is it the same river?

A: Yes.

Q: What if the river froze over and cars started driving on it?

A: Cars would use it as a road.

Q: What’s a word?

A: It’s like a cat.

Q: What’s your favourite word?

A: Dog, I like dogs.

Q: What would happen if you swapped names with your sister?

A: She would call me a different name.

Q: If a prince in a fairy tale gets changed into a frog by a wicked witch, and then changes back again after he gets kissed by his sweetheart, is the frog still the prince?

A: No, he’s a frog.

Q: If England was destroyed in a big explosion, and Liverpool was rebuilt on the other side of the country, would it still be Liverpool?

A: No, it would be a different city.

Q: What’s a book?

A: Something that you’re reading.

Q: If you and your sister go to the library and take out two copies of the Bible, did you take out the same book?

A: Yes.

Q: Imagine your Dad has a ship and he’s sailing around the ocean, and every day a part of the ship falls off and a new part has to be put on it. After a few weeks every single piece of the ship has been replaced, and all the old pieces are collected and put together to make another ship. Which of the two ships is your Dad’s?

A: The first.

Q: What’s water?

A: You drink it.

Q: What’s a house?

A: Something where you live.

Q: If I put lots of books in my house and invited people to take the books, could it be a library?

A: Not if you live in it.

Q: If I say I painted the house brown, where did the paint go?

A: The colours would go over, around the house.

Kate (7;3):

Q: What’s a river?

A: It’s like where people can go fishing, and made of water.

Q: What if the river froze over and cars started driving on it?

A: It’s a road.

Q: What’s a word?

A: It’s in a dictionary?

Q: What’s your favourite word?

A: Red.

Q: How come?

A: Because I like the colour.

Q: Is ‘blah blah’ a word?

A: No, not a real word.

Q: What would happen if we started calling a radiator a toothbrush?

A: It would be different because it’s still white.

Q: If a prince in a fairy tale gets changed into a frog by a wicked witch, and then changes back again after he gets kissed by his sweetheart, is the frog still the prince?

A: Yes.

Q: If England was destroyed in a big explosion, and Liverpool was rebuilt on the other side of the country, would it still be Liverpool?

A: Yes.

Q: If you and your sister go to the library and take out two copies of the Bible, did you take out the same book?

A: Yes.

Q: What’s water?

A: What you drink.

Q: What’s a ship?

A: It’s just like a boat but much bigger.

Q: Imagine your Dad has a ship and he’s sailing around the ocean, and every day a part of the ship falls off and a new part has to be put on it. After a few weeks every single piece of the ship has been replaced, and all the old pieces are collected and put together to make another ship. Which of the two ships is your Dad’s?

A: The new one because it would be much cleaner.

Q: What’s a house?

A: It’s like a big square with glass bits on it for window so you can look out.

Q: If I put lots of books in my house and invited people to take the books, could it be a library?

A: It would just be silly.

Q: If I say ‘I painted the house brown,’ where’s the paint.

A: On the outside, it would be shiny.

Libby (10;5)

Q: What’s a river?

A: A river’s a big, large path full of water that flows.

Q: Imagine a river which suddenly starts to flow backwards, and if it was really polluted with only a small amount of H2O in. Is it still a river?

A: Well, yes it would, because it wouldn’t be rebuilt with concrete.

Q: What’s a word?

A: A word’s just like sounds and phrases.

Q: What’s your favourite word?

A: Love.

Q: How come?

A: I just like it because it has different meanings, like you love your family and all your friends, and there’s another love you can use to love your toys.

Q: What would happen if we started calling a radiator a toothbrush?

A: It’d be silly really, because if you use a radiator as a toothbrush, I mean if you change the name of a radiator to a toothbrush, it’d be weird because it’s not a brush and it’s not for your teeth.

Q: So would you say what you call a thing is – [interruption]

A: - Yes, what you use it for.

Q: If a prince in a fairy tale gets changed into a frog by a wicked witch, and then changes back again after he gets kissed by his sweetheart, is the frog still the prince?

A: Yes because he’s just getting changed from a frog to a person, so he’s still got the same personality.

Q: If England was destroyed in a big explosion, and Liverpool was rebuilt on the other side of the country, would it still be Liverpool?

A: It’d be different because you’d have ... if it was symmetrical it would be alright but if it wasn’t you’d be struggling to get around because you’ve been used to how it was now.

Q: So would it still be Liverpool?

A: Well yes it would because it’s like where the Liverpudlians live.

Q: What’s a book?

A: It’s something you can read and spend your time with.

Q: If you and your sister go to the library and take out two copies of the Bible, did you take out the same book?

A: No because they might change where it’s set and what characters are in it.

Q: Would it be the same story?

A: Yes it would, probably.

Q: What’s water?

A: Water’s a liquid and it can be used as a chemical as well.

Q: What’s a ship?

A: A ship is a large sort of boat where you can have lots of different things on, and it can be used for heavy things.

Q: Imagine your Dad has a ship and he’s sailing around the ocean, and every day a part of the ship falls off and a new part has to be put on it. After a few weeks every single piece of the ship has been replaced, and all the old pieces are collected and put together to make another ship. Which of the two ships is your Dad’s?

A: The one with all the parts, because it’s your parts.

Q: But what if you said ‘this first one is still my ship, I don’t care about the new one’?

A: It’d be fine because I might have spent ages building it so I might want to keep it.

Q: What’s a house?

A: A house is a large building.

Q: If I put lots of books in my house and invited people to take the books, could it be a library?

A: No because it’s your house with book in.

Q: If I say ‘I painted the house brown,’ where’s the paint?

A: I’d say it was on the outside because you can’t really paint over bricks in the rooms.

Lily (11;8)

Q: What’s a river?

A: Fast flowing water.

Q: Imagine a river which suddenly starts to flow backwards, and if it was really polluted with only a small amount of H2O in. Is it still a river?

A: Still a river.

Q: What’s a word?

A: Something that describes something.

Q: What would happen if we started calling a radiator a toothbrush?

A: People would think it’s something else until people got used to it.

Q: What would happen if you and your sister swapped names?

A: I’d get confused.

Q: You’d still be the same person, though?

A: I’d still get confused.

Q: If a prince in a fairy tale gets changed into a frog by a wicked witch, and then changes back again after he gets kissed by his sweetheart, is the frog still the prince?

A: Yes, he’s the same guy in a different form, like reincarnation sort of thing.

Q: If England was destroyed in a big explosion, and Liverpool was rebuilt on the other side of the country, would it still be Liverpool?

A: Yes.

Q: Define ‘book.’

A: Something that you read.

Q: If you and your sister go to the library and take out two copies of the Bible, did you take out the same book?

A: Yes, it’s just ... some Bibles have an interpretation for a younger point of view, but it’s still the same book. Same stories written by the same people.

Q: What if you read the Bible on your laptop but your sister read it in the library? Did you both read the same book?

A: Yes. It’s still a book. You can download it on your laptop.

Q: What’s water?

A: Something that runs, that’s transparent liquid.

Q: Do you know what H2O is?

A: Yes, water.

Q: It’s the same thing?

A: Yes, just a different name.

Q: What’s a ship?

A: Something that floats on water.

Q: Imagine your Dad has a ship and he’s sailing around the ocean, and every day a part of the ship falls off and a new part has to be put on it. After a few weeks every single piece of the ship has been replaced, and all the old pieces are collected and put together to make another ship. Which of the two ships is your Dad’s?

A: Both would be yours, because they both belong to you, because that’s what the definition of ‘yours’ is.

Q: What’s a house?

A: Something that you live in with windows and doors.

Q: If I put lots of books in my house and invited people to take the books, could it be a library?

A: If you wanted it to be.

Q: If I say ‘I painted the house brown,’ where’s the paint?

A: I’d say you painted the outside. If I painted the rooms I’d say I painted my room brown.
